# Association between atherogenic index of plasma and depression in individuals with different glucose metabolism status

**DOI:** 10.3389/fpsyt.2025.1530940

**Published:** 2025-06-03

**Authors:** Chunhui Shi, Change He, Lijie Qin, Weimin Bai

**Affiliations:** ^1^ Department of Endocrinology, The People’s Hospital of Danyang, Danyang Hospital of Nantong University, Danyang, Jiangsu, China; ^2^ Department of Emergency, Henan Provincial People’s Hospital, People’s Hospital of Zhengzhou University, People’s Hospital of Henan University, Zhengzhou, China

**Keywords:** atherogenic index of plasma, depression, diabetes mellitus, glucose metabolism status, CHARLS

## Abstract

**Background:**

The atherogenic index of plasma (AIP) has been implicated in various disease processes, yet its relationship with depression, particularly in the context of differing glucose metabolism status, remains underexplored. This study aimed to investigate the association between AIP and depression in middle-aged and older adults with varying glucose metabolism profiles.

**Methods:**

Data were derived from the China Health and Retirement Longitudinal Study (CHARLS) conducted in 2011 and 2018, encompassing 7,723 participants aged 45 years and above. Depression was defined using a cutoff score of ≥12 on the 10-item Center for Epidemiologic Studies Depression Scale (CESD-10). The primary outcome of interest was incident depression. Logistic regression and restricted cubic spline (RCS) models were applied to assess the relationship between baseline AIP levels and depression risk across distinct glucose metabolism categories.

**Results:**

Elevated AIP was strongly associated with increased odds of depression. In fully adjusted models, a graded relationship was observed, with higher quartiles of AIP corresponding to greater depression risk. Participants in the highest AIP quartile (Q4) had significantly increased odds of depression (odds ratio [OR]: 3.36, 95% confidence interval [CI]: 2.67-4.24, P < 0.001) compared to those in the lowest quartile (Q1). Furthermore, RCS analyses revealed a significant positive association between AIP and incident depression among individuals with prediabetes mellitus (Pre-DM) and diabetes mellitus (DM) (P < 0.001), whereas no such association was found in participants with normal glucose regulation (NGR) (P = 0.086). These findings suggest that glucose metabolism status modifies the relationship between AIP and depression risk.

**Conclusion:**

Higher baseline AIP levels are significantly associated with an increased risk of depression in middle-aged and older adults, with distinct effects modulated by glucose metabolism status. These results highlight the potential utility of AIP as a biomarker for depression risk and suggest that metabolic health should be considered in the development of targeted strategies for depression prevention and intervention.

## 1 Introduction

Depression is a prevalent mental health disorder that imposes a substantial burden on global health and is a significant risk factor for suicide. It is anticipated that depression will become the second leading cause of disease burden worldwide within the next two decades, highlighting the urgency of addressing this public health issue (1). A robust body of literature further substantiates the strong association between depression and various chronic physical conditions (2, 3). Consequently, early identification and diagnosis of depression are essential for timely intervention and recovery.

The atherogenic index of plasma (AIP), calculated as the logarithmic ratio of triglycerides (TG) to high-density lipoprotein cholesterol (HDL-C), is widely utilized to evaluate lipid metabolic disorders (4, 5). Emerging research has implicated dyslipidemia in the etiology and progression of depression, with AIP gaining recognition as a relevant lipid marker in this context (6, 7). Although originally designed to predict the risk of atherosclerosis (8), recent findings have suggested a potential link between AIP and the incidence of depression (9, 10), proposing AIP as a potential biomarker for depressive disorders.

The bidirectional relationship between diabetes mellitus (DM) and depression is well-established, with each condition significantly elevating the risk of the other (11). Insulin resistance (IR), a core feature of DM, is also implicated in the pathophysiology of depression, and may represent a state-dependent metabolic dysfunction in individuals with depression (12, 13). Notably, AIP has been strongly associated with the onset of prediabetes mellitus (Pre-DM) and DM, with elevated AIP levels reflecting the severity of IR (14, 15). Given these links, AIP may serve as a valuable indicator of dysregulated glucose metabolism and a potential predictor of depression in individuals with impaired glucose metabolism, a condition that has also been associated with poorer psychiatric outcomes and treatment response in prior studies (16). However, to date, no studies have comprehensively examined the relationship between AIP and depression across varying glucose metabolism status. Large-scale cohort studies are warranted to elucidate this relationship and to identify novel biomarkers that could facilitate the early detection of depressive symptoms and prompt intervention.

This study, utilizing data from the China Health and Retirement Longitudinal Study (CHARLS), seeks to explore the association between AIP and depression in middle-aged and older adults with differing glucose metabolism status. The findings aim to provide robust population-based evidence to clarify the potential role of AIP as a biomarker for depression.

## 2 Materials and methods

### 2.1 Study population

**Figure 1:**
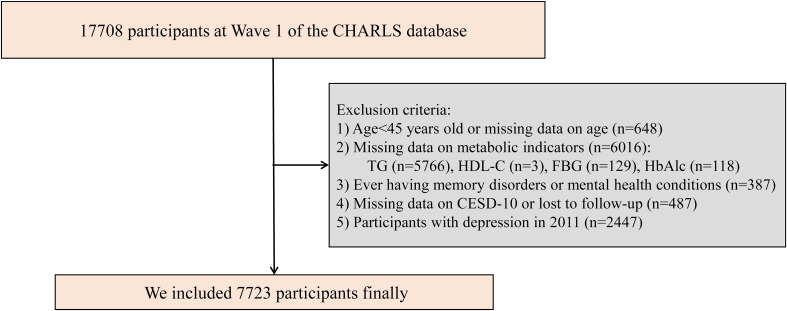
Study flowchart. CHARLS, China Health and Retirement Longitudinal Study; CESD-10, the 10-item Center for Epidemiologic Studies Depression Scale; FBG, fasting blood glucose; HbAlc, glycated hemoglobin; HDL-C, high-density lipoprotein cholesterol; TG, triglycerides.

The CHARLS is a comprehensive national survey aimed at collecting longitudinal health and social data from Chinese individuals aged 45 years and older (http://charls.pku.edu.cn/). The baseline survey was conducted in 2011 and employed a multistage, stratified, probability-proportional-to-size sampling strategy to ensure national representativeness. A total of 17,708 participants from 10,257 households across 450 villages in 150 counties and 28 provinces in China were enrolled. Participants were interviewed face-to-face in their homes using computer-assisted personal interviewing technology. Standardized questionnaires were used to collect comprehensive data on sociodemographic characteristics, lifestyle behaviors, health status, family structure, income and assets, medical insurance, and healthcare utilization. In addition, physical measurements and fasting blood samples were obtained to support biomarker-based analyses. Follow-up surveys have been conducted every two to three years after the baseline. To date, five waves of follow-up surveys have been completed. This study utilized data from the 2011 and 2018 CHARLS surveys, with the 2011 wave serving as the baseline. An initial cohort of 17,708 participants was selected from the CHARLS database. After applying specific exclusion criteria, we excluded individuals: (1) aged <45 years or with missing age data (n = 648); (2) with missing data on any of the following metabolic indicators: TG (n = 5,766), HDL-C (n = 3), fasting blood glucose (FBG) (n = 129), or glycated hemoglobin (HbA1c) (n = 118), resulting in a total of 6,016 exclusions; (3) with a history of memory disorders or mental health conditions (n = 387); (4) with missing data on the 10-item Center for Epidemiologic Studies Depression Scale (CESD-10) or lost to follow-up in 2018 (n = 487); (5) who had depression in 2011 (n = 2,447). As a result, a total of 7,723 participants met the inclusion criteria and were included in the final analysis (**Figure 1**). These participants were stratified into quartiles based on their baseline AIP and followed up through 2018.

The CHARLS study received ethical approval from the Institutional Review Board of Peking University (IRB00001052-11015). Informed consent was obtained from all participants through signed consent forms prior to their participation.

### 2.2 Measurement of depression and atherogenic index of plasma

Depressive was assessed using the CESD-10, a validated instrument commonly employed in large-scale population studies to measure depression (17). The CESD-10 includes items such as “bothered by little things,” “felt depressed,” and “could not get going.” A total score of 12 or above was defined as indicative of depression (18).

Lipid levels, including TG and HDL-C, were measured using standard enzymatic colorimetric assays (19). The AIP was calculated as the logarithmic ratio of TG to HDL-C, expressed in mg/dL.

### 2.3 Data collection and definitions

In this study, we classified variables into five main categories: (1) sociodemographic factors, encompassing age, gender, marital status, residence, and education level; (2) anthropometric measurements, including body mass index (BMI); (3) health behaviors, such as smoking and drinking status; (4) medical history; and (5) laboratory parameters, which included total cholesterol (TC), TG, HDL-C, low-density lipoprotein cholesterol (LDL-C), FBG, and HbA1c. DM was defined as FBG ≥ 126 mg/dL, HbA1c ≥ 6.5%, and/or a self-reported physician diagnosis of DM or the use of glucose-lowering medications (20). Pre-DM was defined as FBG between 100 and 125 mg/dL or HbA1c between 5.7% and 6.4%, in accordance with established diagnostic criteria. Participants without DM or Pre-DM were categorized as having normal glucose regulation (NGR). Hypertension was defined by self-reported hypertension, the use of antihypertensive therapy, and/or systolic blood pressure (SBP) ≥ 140 mmHg or diastolic blood pressure (DBP) ≥ 90 mmHg. Dyslipidemia was identified based on self-reported physician diagnosis, the use of lipid-lowering medications, or meeting any of the following criteria: TC ≥ 240 mg/dL, TG ≥ 150 mg/dL, HDL-C < 40 mg/dL, or LDL-C ≥ 160 mg/dL (21). BMI was calculated using the standard formula: weight (kg)/height² (m²).

### 2.4 Covariates

To account for potential confounding factors, we considered a comprehensive set of covariates, including age, gender, BMI, marital status, residence, educational level, health status, smoking and alcohol consumption, presence of chronic diseases, hypertension, LDL-C, TC, cognitive function score, and CESD-10 score derived from the 2011 survey.

### 2.5 Statistical analysis

Descriptive statistics were used to summarize the data, with variables presented as mean ± standard deviation (SD), median (interquartile range), or frequency and percentage, depending on the data type. Categorical variables were analyzed using the chi-square test, while continuous variables were assessed using one-way analysis of variance (ANOVA) or the Kruskal-Wallis test for non-normally distributed data.

To investigate the association between the AIP and depression, we employed logistic regression analysis, reporting the results as adjusted odds ratio (OR) with 95% confidence interval (CI). This approach was appropriate given that depression was assessed only at discrete follow-up time points, without precise information on the timing of onset. Model 1 was based on univariate logistic regression; Model 2 adjusted for age and gender, and Model 3 further adjusted for a wider range of variables: age, gender, BMI, marital status, residence, educational level, health status, smoking status, alcohol consumption, chronic diseases, hypertension, LDL-C, TC, cognitive function score, and CESD-10 score in 2011. Restricted cubic spline (RCS) regression was used to examine the dose-response relationship between AIP and depression, with three knots placed at the 25th, 50th (median), and 75th percentiles of the AIP levels. We further examined the linear or nonlinear association between baseline AIP levels and depression risk within the NGR, Pre-DM, and DM subgroups using RCS analysis.

Additionally, Cox regression was performed as a sensitivity analysis to assess the robustness of the primary results. Subgroup analyses were conducted to assess the relationship between AIP and depression risk across various demographic and clinical subgroups, stratified by age (45–60 years and ≥ 60 years), gender (male and female), BMI (< 24 kg/m^2^ and ≥ 24 kg/m^2^), residence (urban and rural), and hypertension (yes and no). All statistical analyses were conducted using R version 4.2.2 (R Foundation for Statistical Computing, Vienna, Austria), with a two-tailed P-value of < 0.05 considered statistically significant.

## 3 Results

### 3.1 Baseline characteristics

The study cohort comprised 7,723 participants with a mean age of 58.9 ± 9.3 years, of which 50% were female (n = 3,858). Participants were stratified into four groups according to AIP quartiles: Q1 (n = 1,931), Q2 (n = 1,930), Q3 (n = 1,931), and Q4 (n = 1,931). The Q4 group exhibited a higher prevalence of dyslipidemia and elevated triglycerides. A majority of participants resided in rural areas (60.6%) and lacked formal education (43.3%). Additionally, 65.7% had a history of chronic diseases, and 31.9% were current smokers. Significant differences were observed among the quartiles with respect to age, gender, BMI, marital status, residence, education level, smoking status, alcohol consumption, hypertension, dyslipidemia, coronary heart disease, stroke, lipid parameters (TC, TG, HDL-C, LDL-C), FBG, cognitive function scores, and glucose metabolic states (NGR, Pre-DM, DM) (all P < 0.05). Detailed baseline characteristics are summarized in **Table 1**.

**Table 1:** Baseline characteristics of participants categorized by AIP quartiles.

Characteristic	Overall (n = 7723)	AIP				
		Quartile 1 (n = 1931)	Quartile 2 (n = 1930)	Quartile 3 (n = 1931)	Quartile 4 (n = 1931)	P value
Age, mean ± SD, years	58.9 ± 9.3	59.6 ± 9.8	59.2 ± 9.5	58.7 ± 9.2	58.1 ± 8.7	<0.001
Female, n (%)	3858 (50.0)	865 (44.8)	955 (49.5)	1035 (53.6)	1003 (51.9)	< 0.001
BMI, kg/m^2^	24.39 (3.41)	22.00 (3.26)	25.07 (3.88)	24.84 (3.33)	25.70 (3.95)	0.004
Marital status, n (%)						0.018
Married	6959 (90.1)	1723 (89.2)	1722 (89.2)	1740 (90.1)	1774 (91.9)	
Others	764 (9.9)	208 (10.8)	208 (10.8)	191 (9.9)	157 (8.1)	
Residence, n (%)						<0.001
Rural	4678 (60.6)	1321 (68.4)	1218 (63.1)	1120 (58.0)	1019 (52.8)	
Urban	3045 (39.4)	610 (31.6)	712 (36.9)	811 (42.0)	912 (47.2)	
Education level, n (%)						<0.001
No formal education	3342 (43.3)	903 (46.8)	850 (44.1)	812 (42.1)	777 (40.3)	
Primary school	1703 (22.1)	431 (22.3)	438 (22.7)	423 (22.0)	411 (21.3)	
Middle or high school	1702 (22.1)	401 (20.8)	412 (21.4)	435 (22.6)	454 (23.5)	
College or above	967 (12.5)	194 (10.1)	228 (11.8)	257 (13.3)	288 (14.9)	
Health, n (%)						0.063
Poor	160 (2.1)	36 (1.9)	40 (2.1)	46 (2.4)	38 (2.0)	
Fair	1383 (17.9)	336 (17.4)	328 (17.0)	374 (19.4)	345 (17.9)	
Good	4120 (53.4)	1056 (54.6)	1056 (54.7)	1020 (52.8)	988 (51.1)	
Very good and above	2055 (26.6)	503 (26.1)	504 (26.2)	489 (25.4)	559 (29.0)	
Smoking status, n (%)						<0.001
Never or former	5260 (68.1)	1240 (64.2)	1281 (66.4)	1369 (70.9)	1370 (70.9)	
Current	2461 (31.9)	690 (35.8)	648 (33.6)	562 (29.1)	561 (29.1)	
Drinking status, n (%)						<0.001
Never or former	5026 (65.1)	1128 (58.4)	1263 (65.4)	1335 (69.1)	1300 (67.3)	
Current	2697 (34.9)	803 (41.6)	667 (34.6)	596 (30.9)	631 (32.7)	
Hypertension, n (%)	3548 (46.0)	744 (38.6)	807 (41.9)	954 (49.5)	1043 (54.1)	< 0.001
Dyslipidemia, n (%)	3965 (51.3)	680 (35.2)	787 (40.8)	1007 (52.1)	1491 (77.2)	< 0.001
CHD, n (%)	822 (10.7)	159 (8.3)	171 (8.9)	218 (11.3)	274 (14.2)	<0.001
Stroke, n (%)	144 (1.9)	24 (1.2)	36 (1.9)	34 (1.8)	50 (2.6)	0.021
Chronic diseases, n (%)	5071 (65.7)	1176 (60.9)	1223 (63.4)	1323 (68.5)	1349 (69.9)	<0.001
TC, mean ± SD, mg/dL	193.21 ± 39.05	187.48 ± 34.80	188.81 ± 37.23	194.45 ± 37.52	202.08 ± 44.31	<0.001
TG, median (IQR), mg/dL	106.19 (75.23, 158.42)	62.00 (52.23, 72.61)	89.82 (78.84, 103.25)	127.16 (110.28, 146.80)	215.37 (175.64, 288.12)	< 0.001
HDL-C, mean ± SD, mg/dL	50.59 ± 15.11	66.20 ± 13.94	53.42 ± 10.01	46.38 ± 8.77	36.37 ± 8.39	<0.001
LDL-C, mean ± SD, mg/dL	116.20 ± 35.16	111.27 ± 30.62	119.14 ± 33.30	123.74 ± 34.35	110.62 ± 40.02	<0.001
FBG, mean ± SD, mg/dL	110.37 ± 36.86	102.30 ± 24.50	106.37 ± 32.20	109.56 ± 35.32	123.26 ± 47.99	<0.001
Cognitive function score	12.40 (3.40)	12.01 (3.51)	12.35 (3.46)	12.51 (3.27)	12.72 (3.33)	<0.001
CESD-10 score in 2011	5.56 (3.57)	5.47 (3.59)	5.48 (3.56)	5.51 (3.58)	5.76 (3.53)	0.037
CESD-10 score in 2018	6.09 (6.09)	4.64 (5.21)	5.61 (5.75)	6.19 (6.34)	7.92 (6.51)	<0.001
GMS, n (%)						<0.001
NGR	3166 (41.0)	979 (50.7)	880 (45.6)	775 (40.1)	532 (27.6)	
Pre-DM	3403 (44.1)	794 (41.1)	829 (43.0)	865 (44.8)	915 (47.4)	
DM	1154 (14.9)	158 (8.2)	221 (11.5)	291 (15.1)	484 (25.1)	

Continuous variables were shown in mean (SD) and categorical variables were shown in percentages.

AIP, atherogenic index of plasma; BMI, body mass index; CESD-10, the 10-item Center for Epidemiologic Studies Depression Scale; CHD, coronary heart disease; DM, diabetes mellitus; FBG, fasting blood glucose; GMS, glucose metabolic states; HDL-C, high-density lipoprotein cholesterol; IQR, inter quartile range; LDL-C, low-density lipoprotein cholesterol; NGR, normal glucose regulation; Pre-DM, prediabetes mellitus; SD, standard deviation; TC, total cholesterol; TG, triglycerides.

### 3.2 Association between AIP and depression

**Figure 2:**
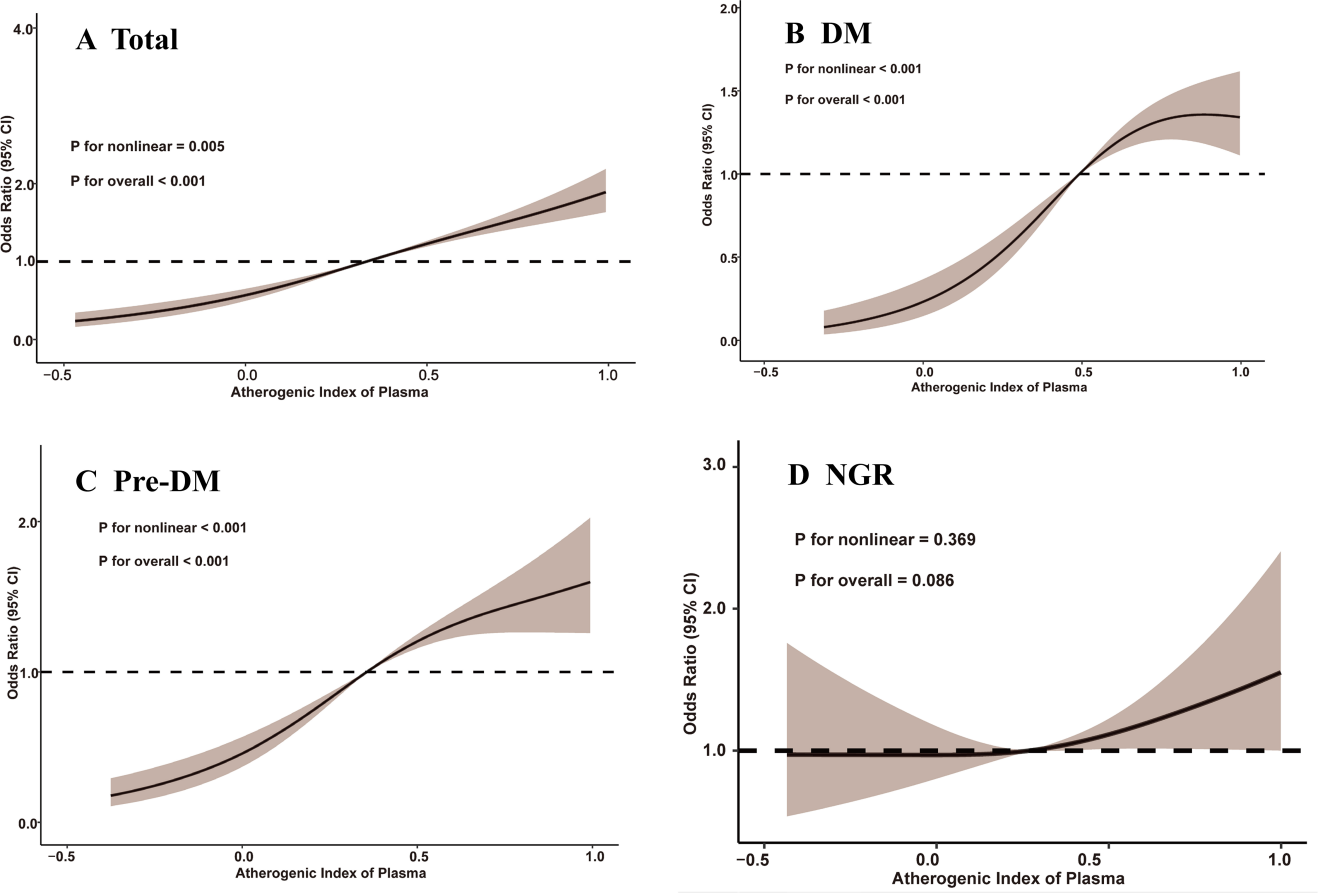
Restricted cubic spline analysis of the association between atherogenic index of plasma and depression. **(A)** total participants; **(B)** participants with DM; **(C)** participants with Pre-DM. **(D)** participants with NGR. CI, confidence interval; DM, diabetes mellitus; NGR, normal glucose regulation; Pre-DM, prediabetes mellitus.

Between 2011 and 2018, a total of 1,311 participants (17.0%) developed depression, accompanied by an overall upward trend in CESD-10 scores. When stratified by quartiles of AIP, the proportions of participants who developed depression were 10.0% (n = 194) in Q1, 13.6% (n = 263) in Q2, 18.6% (n = 359) in Q3, and 25.6% (n = 495) in Q4. After adjusting for potential confounders, a significant association between AIP quartiles and depression risk was identified. Compared to participants in Q1, those in Q3 and Q4 exhibited a markedly higher risk of depression (OR: 2.33, 95% CI: 1.85-2.94, P < 0.001; OR: 3.36, 95% CI: 2.67-4.24, P < 0.001). Furthermore, continuous analysis revealed that each one-unit increase in AIP was associated with a 312% increase in the risk of depression in Model 3 (**Table 2**). RCS analysis corroborated a significant nonlinear dose-response relationship between AIP and depression risk (P for overall < 0.001; P for nonlinear = 0.005), as illustrated in **Figure 2**.

**Table 2:** The association between AIP and the risk of depression.

Categories	Event, n (%)	Model 1 ^a^		Model 2 ^b^		Model 3 ^c^	
		OR (95% CI)	P value	OR (95% CI)	P value	OR (95% CI)	P value
Per 1 unit increase	1311 (17.0%)	3.23 (2.73-3.82)	<0.001	3.15 (2.66-3.74)	<0.001	4.12 (3.20-5.32)	<0.001
Quartile 1	194 (10.0%)	Ref.		Ref.		Ref.	
Quartile 2	263 (13.6%)	1.41 (1.16-1.72)	<0.001	1.38 (1.13-1.68)	0.001	1.67 (1.32-2.13)	<0.001
Quartile 3	359 (18.6%)	2.04 (1.70-2.47)	<0.001	1.96 (1.62-2.37)	<0.001	2.33 (1.85-2.94)	<0.001
Quartile 4	495 (25.6%)	3.09 (2.58-3.70)	<0.001	2.98 (2.49-3.57)	<0.001	3.36 (2.67-4.24)	<0.001

AIP, atherogenic index of plasma; CI, confidence interval; OR, odds ratio.

a Unadjusted model.

b Adjusted for age and gender.

c Adjusted for age, gender, body mass index, marital status; residence, educational level, health, smoking status, drinking status, chronic diseases, hypertension, low-density lipoprotein cholesterol, total cholesterol, cognitive function score, and CESD-10 score in 2011.

### 3.3 Associations between AIP and depression moderated by glucose metabolic states

Among participants with different glucose metabolic states, 327 individuals (10.3%) in the NGR group, 691 individuals (20.3%) in the Pre-DM group, and 293 individuals (25.4%) in the DM group developed depression by the end of follow-up. As shown in **Table 3**, Model 3 revealed that higher AIP quartiles were significantly associated with an elevated risk of depression among Pre-DM and DM participants relative to Q1. In the Pre-DM group, the ORs were 2.00 (95% CI: 1.41-2.87) for Q2, 3.22 (95% CI: 2.30-4.55) for Q3, and 4.25 (95% CI: 3.04-5.99) for Q4, with a p-value of 0.001. Among DM participants, the ORs were 1.84 (95% CI: 0.87-4.13) for Q2, 3.12 (95% CI: 1.56-6.72) for Q3, and 4.73 (95% CI: 2.43-9.00) for Q4, with a p-value of 0.001. RCS analysis demonstrated a nonlinear relationship between baseline AIP and depression risk in both Pre-DM and DM participants (Pre-DM: P for nonlinear < 0.001; DM: P for nonlinear < 0.001). Conversely, no significant dose-response relationship was observed between AIP and depression risk in the NGR group (**Figure 2**).

**Table 3:** The association between AIP and the risk of depression according to glucose metabolic states.

Categories	Event, n (%)	Model 1 ^a^		Model 2 ^b^		Model 3 ^c^		P-interaction
		OR (95% CI)	P value	OR (95% CI)	P value	OR (95% CI)	P value	
NGR								0.004
Per 1 unit increase	327 (10.3%)	1.47 (0.99-2.16)	0.500	1.43 (0.97-2.10)	0.071	1.55 (0.94-2.54)	0.085	
Quartile 1	93 (9.5%)	Ref.		Ref.		Ref.		
Quartile 2	91 (10.3%)	1.10 (0.81-1.49)	0.544	1.07 (0.79-1.46)	0.050	1.28 (0.88-1.87)	0.202	
Quartile 3	82 (10.6%)	1.13 (0.82-1.54)	0.453	1.08 (0.79-1.48)	0.634	1.17 (0.79-1.75)	0.433	
Quartile 4	61 (11.5%)	1.23 (0.87-1.73)	0.228	1.21 (0.85-1.70)	0.280	1.27 (0.81-1.97)	0.286	
Pre-DM								
Per 1 unit increase	691 (20.3%)	3.41 (2.65-4.40)	<0.001	3.25 (2.51-4.21)	<0.001	4.83 (3.36-6.99)	<0.001	
Quartile 1	87 (12.2%)	Ref.		Ref.		Ref.		
Quartile 2	134 (16.2%)	1.57 (1.18-2.10)	0.002	1.55 (1.16-2.07)	0.003	2.00 (1.41-2.87)	<0.001	
Quartile 3	199 (23.0%)	2.43 (1.85-3.20)	<0.001	2.33 (1.78-3.08)	<0.001	3.22 (2.30-4.55)	<0.001	
Quartile 4	271 (29.6%)	3.42 (2.64-4.47)	<0.001	3.25 (2.50-4.26)	<0.001	4.25 (3.04-5.99)	<0.001	
DM								
Per 1 unit increase	293 (25.4%)	2.59 (1.89-3.56)	<0.001	2.41 (1.75-3.32)	<0.001	4.13 (2.28-7.68)	<0.001	
Quartile 1	14 (8.9%)	Ref.		Ref.		Ref.		
Quartile 2	38 (17.2%)	2.14 (1.14-4.22)	0.022	1.98 (1.05-3.94)	0.040	1.84 (0.87-4.13)	0.121	
Quartile 3	78 (26.8%)	3.77 (2.11-7.17)	<0.001	3.46 (1.93-6.62)	<0.001	3.12 (1.56-6.72)	0.002	
Quartile 4	163 (33.7%)	5.22 (3.02-9.72)	<0.001	4.71 (2.71-8.79)	<0.001	4.73 (2.43-9.00)	<0.001	

AIP, atherogenic index of plasma; CI, confidence interval; DM, diabetes mellitus; NGR, normal glucose regulation; OR, odds ratio; Pre-DM, prediabetes mellitus.

a Unadjusted model.

b Adjusted for age and gender.

c Adjusted for age, gender, body mass index, marital status; residence, educational level, health, smoking status, drinking status, chronic diseases, hypertension, low-density lipoprotein cholesterol, total cholesterol, cognitive function score, and CESD-10 score in 2011.

Bold values indicate subgroup headings defined by glucose metabolism status: NGR, Pre-DM, and DM.

### 3.4 Subgroup and sensitivity analyses

Subgroup analyses based on age, gender, BMI, residence, and hypertension showed that the proportion of participants who developed depression increased progressively with higher AIP quartiles. These analyses demonstrated consistent patterns across all subgroups, with no significant interactions observed (all P- interaction > 0.05). In each subgroup, participants in the highest AIP quartile (Q4) consistently exhibited a significantly higher risk of depression (**Table 4**). The sensitivity analysis yielded results consistent with the main findings, showing a stable and positive association between higher AIP levels and increased depression risk. The pattern across glucose metabolism status groups was also consistent with that observed in the primary analysis (**Supplementary Tables 1**, **2**).

**Table 4:** Subgroup and interaction analysis between the AIP and depression across various subgroups.

Subgroups	AIP	Event (%)	OR (95% CI)	P value		AIP	Event (%)	OR (95% CI)	P value	P-interaction
Age										0.479
<60 years	Q1	114 (10.7)	Ref.		≥60 years	Q1	80 (9.2)	Ref.		
	Q2	153 (14.2)	1.59 (1.16-2.18)	0.004		Q2	110 (12.9)	1.82 (1.25-2.66)	0.002	
	Q3	206 (18.7)	2.10 (1.56-2.86)	<0.001		Q3	153 (18.5)	2.72 (1.89-3.96)	<0.001	
	Q4	326 (28.1)	3.61 (2.70-4.88)	<0.001		Q4	169 (21.9)	2.95 (2.04-4.34)	<0.001	
Gender										0.670
Female	Q1	112 (12.9)	Ref.		Male	Q1	82 (7.7)	Ref.		
	Q2	155 (16.2)	1.62 (1.17-2.26)	0.004		Q2	108 (11.1)	1.74 (1.22-2.49)	0.002	
	Q3	230 (22.2)	2.29 (1.68-3.15)	<0.001		Q3	129 (14.4)	2.40 (1.70-3.42)	<0.001	
	Q4	306 (30.5)	3.49 (2.56-4.80)	<0.001		Q4	189 (20.4)	3.24 (2.30-4.59)	<0.001	
BMI										0.702
<24 kg/m^2^	Q1	132 (9.8)	Ref.		≥24 kg/m^2^	Q1	44 (11.6)	Ref.		
	Q2	152 (13.9)	1.63 (1.22-2.18)	<0.001		Q2	90 (14.6)	1.75 (1.12-2.79)	0.017	
	Q3	160 (18.8)	2.34 (1.75-3.13)	<0.001		Q3	155 (18.5)	2.36 (1.55-3.68)	<0.001	
	Q4	145 (22.8)	3.06 (2.25-4.18)	<0.001		Q4	291 (28.1)	3.64 (2.42-5.65)	<0.001	
Residence										0.800
Urban	Q1	51 (8.4)	Ref.		Rural	Q1	143 (10.8)	Ref.		
	Q2	67 (9.4)	1.47 (0.93-2.38)	0.105		Q2	196 (16.1)	1.76 (1.33-2.33)	<0.001	
	Q3	118 (14.5)	2.36 (1.55-3.69)	<0.001		Q3	241 (21.5)	2.30 (1.75-3.05)	<0.001	
	Q4	188 (20.6)	3.01 (1.99-4.67)	<0.001		Q4	307 (30.1)	3.61 (2.73-4.79)	<0.001	
Hypertension										0.146
No	Q1	120 (10.1)	Ref.		Yes	Q1	73 (9.8)	Ref.		
	Q2	162 (14.5)	1.92 (1.40-2.64)	<0.001		Q2	101 (12.5)	1.43 (0.98-2.09)	0.063	
	Q3	178 (18.3)	2.49 (1.82-3.42)	<0.001		Q3	180 (18.9)	2.25 (1.59-3.21)	<0.001	
	Q4	207 (23.4)	3.09 (2.24-4.28)	<0.001		Q4	288 (27.6)	3.69 (2.64-5.23)	<0.001	

Model adjusted for age, gender, body mass index, marital status; residence, educational level, health, smoking status, drinking status, chronic diseases, hypertension, low-density lipoprotein cholesterol, total cholesterol, cognitive function score, and CESD-10 score in 2011. AIP, atherogenic index of plasma; BMI, body mass index; OR, odds ratios; CI, confidence intervals.

## 4 Discussion

This study offers new evidence on the relationship between AIP levels and depression in middle-aged and older adults with varying glucose metabolic statuses. Our analysis reveals a positive correlation between elevated AIP levels and the risk of depression, characterized by a nonlinear association. This effect was particularly marked in individuals with abnormal glucose metabolism, including those with Pre-DM and DM. These findings suggest that baseline AIP may serve as a valuable biomarker for identifying individuals at higher risk of depression, particularly in those with glucose metabolism disorders. Additionally, maintaining lower AIP levels could offer a strategic target for the primary prevention of depression in these populations.

Depression, a prevalent psychological disorder, manifests as persistent feelings of sadness, diminished interest in activities, and cognitive, behavioral, or physical symptoms (22, 23). It is a significant contributor to morbidity, increasing the risk of suicide and susceptibility to various diseases, particularly cardiovascular disease (CVD) (24). Previous studies have consistently demonstrated a close association between CVD and depression, with both conditions sharing overlapping pathophysiological mechanisms (25, 26). Epidemiological data consistently highlight an increased risk of cardiovascular events in individuals with depressive symptoms (27).

As a marker linked to atherosclerosis and inflammation, AIP has emerged as a potential biomarker with significant implications for predicting both CVD and depression (28, 29). Notably, research by Sandra et al. found that individuals with depression exhibited significantly elevated AIP levels (5). A large study using data from the National Health and Nutrition Examination Survey (NHANES) from 2005 to 2018, encompassing 12,453 participants, demonstrated an L-shaped relationship between AIP and depression, with a critical threshold at 0.289 (10). A Above this threshold, higher AIP levels were associated with a significantly increased risk of depression. Similar findings were reported by Ye et al., who observed that elevated AIP levels correlated with greater susceptibility to depressive symptoms (9). A retrospective cross-sectional study from Brazil also demonstrated a relationship between elevated AIP levels and an increased prevalence of depression (5). However, this study had limitations, including the exclusion of individuals aged 65 and older and a small sample size of 331 participants, limiting the generalizability of the findings. Given that older adults are more prone to lipid metabolism disorders and depression due to reduced metabolic rates and social interactions, these limitations warrant further investigation (30). Our study, which included 7,723 participants aged 45 years and older, provides robust evidence of a significant positive association between elevated AIP levels and depressive symptoms. These findings underscore the importance of AIP as a potential predictor of depression (9). Further research elucidating the role of AIP in mood disorders and its interplay with cardiovascular health could advance our understanding of the biological pathways linking these conditions.

Importantly, our study revealed significant differences in the association between the AIP and depressive symptoms among individuals with DM and Pre-DM, whereas no significant association was observed in the normoglycemic group. DM is characterized by disruptions in glucose and lipid metabolism, predisposing individuals to atherogenic dyslipidemia and heightened cardiovascular risk (31). Elevated AIP levels have been linked to insulin resistance, and individuals with DM commonly exhibit higher AIP values (32). Consequently, the coexistence of DM may exacerbate the atherogenic effects associated with elevated AIP levels, thereby contributing to the onset and progression of depressive symptoms. Previous research has established DM as a major risk factor for both CVD and depression (33, 34). A large-scale cross-sectional survey identified a positive correlation between elevated AIP levels and an increased risk of Pre-DM and DM (35), while a separate prospective cohort study demonstrated that major depressive disorder increases the likelihood of DM-related complications (36), However, no studies to date have confirmed the predictive value of baseline AIP levels for depression in individuals with glucose metabolism disorders. Our findings indicate that elevated baseline AIP levels are associated with new-onset depression in Pre-DM and DM patients. In contrast, the RCS curve in the NGR group remained relatively flat, indicating no clear association between AIP and depression. Clinically, this may suggest that among individuals with preserved glucose homeostasis, the impact of lipid-derived atherogenic burden as measured by AIP on neuropsychiatric outcomes such as depression is less pronounced, potentially due to a lower level of systemic metabolic disturbance and inflammatory activation. Therefore, measuring AIP levels in middle-aged and older individuals with abnormal glucose metabolism may have clinical significance, as AIP could potentially serve as a biomarker to predict and identify the risk of depression.

The pathophysiology underlying the relationship between AIP and depression is complex and remains poorly understood, though several potential mechanisms may be involved. The association between AIP and depression could be partially attributed to shared pathways, such as inflammation, oxidative stress, and endothelial dysfunction, all of which are implicated in the progression of both depression and CVD (37, 38). Studies have demonstrated that depression is often accompanied by elevated levels of pro-inflammatory markers, including C-reactive protein and interleukin-6 (39, 40). which not only contribute to the development of depression but also increase the risk of lipid metabolism disorders (41, 42). Dietary factors, particularly high-carbohydrate and high-fat intake, can exacerbate lipid metabolism dysregulation, leading to increased oxidative stress (43). Under physiological conditions, oxidative stress induces the production of reactive oxygen species, which, in turn, stimulate the production of antioxidants to regulate oxidative levels (44). However, persistent oxidative stress may overwhelm these regulatory mechanisms, ultimately leading to sustained inflammation and depression (45). Furthermore, excessive lipid intake has been associated with glial cell accumulation, which can impair hippocampal neurons and elevate the risk of depression (46). These biological processes may result in structural changes in the vasculature and brain, potentially exacerbating depressive symptoms or facilitating the development of CVD.

Our second key finding is the significant interaction between AIP and glucose metabolism status, indicating that the association between AIP and depression is particularly pronounced in individuals with Pre-DM and DM. Several mechanisms may underlie this relationship. Dyslipidemia, through various pathways, can impair pancreatic function and reduce insulin sensitivity, thereby exacerbating the progression of Pre-DM and DM (47, 48). Supporting this, a cross-sectional study demonstrated a strong correlation between elevated AIP levels, increased risk of insulin resistance, and the onset of DM (32). Approximately half of DM patients, both fasting and postprandial, present with elevated triglycerides or reduced HDL-C levels, a pattern frequently observed in individuals with insulin resistance or impaired glucose tolerance (49). Elevated triglyceride concentrations increase free fatty acid levels, impairing insulin sensitivity and contributing to abnormal glucose metabolism (50). In recent years, the relationship between glucose metabolism status and depression has garnered increasing attention. Evidence from psychiatric populations has shown that impaired glucose metabolism may be linked to poorer treatment outcomes, further underscoring the relevance of metabolic status in shaping depression trajectories (16). The bidirectional nature of the DM-depression link is well-established: DM is associated with a 20% increased risk of depression, while depression confers a 60% increased risk of developing DM (51). An independent prospective cohort study further reported that major depressive disorder significantly increases the likelihood of DM-related complications (36). Various biological mechanisms have been proposed to explain the DM-depression association across the life course, including the activation of innate immunity, dysregulation of the acute-phase inflammatory response, chronic dysfunction of the hypothalamic-pituitary-adrenal axis, circadian rhythm disturbances, and insulin resistance (52, 53). The coexistence of DM may exacerbate the atherogenic effects of elevated AIP, thereby contributing to the onset and progression of depressive symptoms.

Given this context, effectively addressing dyslipidemia and maintaining an optimal lipid profile in patients with abnormal glucose metabolism is crucial for the prevention and treatment of depressive symptoms. Our study suggests that AIP may be a valuable biomarker for predicting depression risk in middle-aged and older Chinese adults with glucose metabolism disorders, as we observed a significant association between elevated AIP levels and depression risk in Pre-DM and DM participants. These findings underscore the importance of optimizing cardiovascular health in managing depression among diabetic individuals and provide new perspectives for developing risk stratification and intervention strategies for mental health disorders. Given its simplicity, cost-effectiveness, and widespread availability in clinical settings, AIP screening could be feasibly integrated into routine metabolic assessments among high-risk individuals, particularly those with impaired glucose regulation. Physiological indices like AIP offer quantifiable measures for assessing depressive symptoms, enabling targeted interventions that could contribute to the primary prevention of depression. Furthermore, continued research is necessary to identify additional risk factors that can predict depression, facilitating early preventive strategies.

This study has several limitations. First, the analysis focused solely on baseline AIP levels without accounting for longitudinal variations in AIP over the course of follow-up. This limits the capacity to infer the dynamic relationship between AIP and depressive symptoms. Second, although several potential confounders were adjusted for in the analysis, the potential influence of residual or unmeasured confounding factors cannot be entirely excluded. In particular, information on lipid-lowering medications such as statins was not available in the current CHARLS dataset, which may have influenced AIP levels but could not be accounted for in this analysis. Future studies should aim to incorporate detailed medication data to more accurately control for this potential confounder. Third, the possibility that depressive symptoms may influence metabolic parameters such as lipid levels cannot be fully excluded. Additionally, the CHARLS database lacks precise information on the timing of depression onset, which limited our ability to perform time-to-event analyses based on exact incidence dates. Future studies with more frequent follow-up assessments are needed to clarify the temporal nature of this relationship. Lastly, the study population was restricted to individuals of Chinese ethnicity, and the generalizability of the findings to other ethnic groups or populations remains uncertain. Further research should strive to account for potential confounding variables and examine these relationships in more diverse populations to enhance the broader applicability of the results.

## 5 Conclusion

This study identified a significant association between elevated AIP levels and an increased risk of depression in middle-aged and older adults, particularly those with Pre-DM and DM. AIP holds potential as a biomarker for depression risk stratification in individuals with glucose metabolism disorders.

## Data Availability

Publicly available datasets were analyzed in this study. This data can be found here: The data is sourced from the publicly accessible CHARLS database (http://charls.pku.edu.cn/). The datasets used during the current study are available from the corresponding author on reasonable request.

## Supplementary material

Supplementary Table 1 The association between AIP index (quartiles) and the risk of depression. AIP: atherogenic index of plasma; CI: confidence interval; HR: hazard ratios. ^a^ Unadjusted model ^b^ Adjusted for age and gender ^c^ Adjusted for age, gender, body mass index, marital status; residence, educational level, health, smoking status, drinking status, chronic diseases, hypertension, low-density lipoprotein cholesterol, total cholesterol, cognitive function score, and CESD-10 score in 2011.

Supplementary Table 2 The association between AIP and the risk of depression according to glucose metabolic states. AIP: atherogenic index of plasma; CI: confidence interval; DM: diabetes mellitus; HR: hazard ratios; NGR: normal glucose regulation; Pre-DM: prediabetes mellitus. ^a^ Unadjusted model ^b^ Adjusted for age and gender ^c^ Adjusted for age, gender, body mass index, marital status; residence, educational level, health, smoking status, drinking status, chronic diseases, hypertension, low-density lipoprotein cholesterol, total cholesterol, cognitive function score, and CESD-10 score in 2011.
