# Supplementary material for: Association between atherogenic index of plasma and depression in individuals with different glucose metabolism status
Source: Front Psychiatry. 2025 Jun 3;16:1530940. doi: 10.3389/fpsyt.2025.1530940 (PMC12170628; doi:10.3389/fpsyt.2025.1530940)
Supplement: Supplementary Table 1 — The association between AIP index (quartiles) and the risk of depression. AIP: atherogenic index of plasma; CI: confidence interval; HR: hazard ratios. a Unadjusted model b Adjusted for age and gender c Adjusted for age, gender, body mass index, marital status; residence, educational level, health, smoking status, drinking status, chronic diseases, hypertension, low-density lipoprotein cholesterol, total cholesterol, cognitive function score, and CESD-10 score in 2011. [file Table1.docx]

**Supplementary Table 1 The association between AIP index (quartiles) and the risk of depression**

| Categories | Event, n (%) | Model 1 ^a^ | | Model 2 ^b^ | | Model 3 ^c^ | |
| --- | --- | --- | --- | --- | --- | --- | --- |
|  |  | HR (95% CI) | P value | HR (95% CI) | P value | HR (95% CI) | P value |
| Per 1 unit increase | 1311 (17.0%) | 2.73 (2.38-3.14) | <0.001 | 2.68 (2.32-3.08) | <0.001 | 3.40 (2.73-4.24) | <0.001 |
| Quartile 1 | 194 (10.0%) | Ref. |  | Ref. |  | Ref. |  |
| Quartile 2 | 263 (13.6%) | 1.38 (1.15-1.67) | <0.001 | 1.35 (1.12-1.63) | 0.001 | 1.61 (1.28-2.01) | <0.001 |
| Quartile 3 | 359 (18.6%) | 1.94 (1.63-2.31) | <0.001 | 1.86 (1.56-2.21) | <0.001 | 2.16 (1.74-2.67) | <0.001 |
| Quartile 4 | 495 (25.6%) | 2.79 (2.36-3.29) | <0.001 | 2.69 (2.28-3.17) | <0.001 | 2.95 (2.39-3.63) | <0.001 |

AIP: atherogenic index of plasma; CI: confidence interval; HR: hazard ratios.

^a^ Unadjusted model

^b^ Adjusted for age and gender

^c^ Adjusted for age, gender, body mass index, marital status; residence, educational level, health, smoking status, drinking status, chronic diseases, hypertension, low-density lipoprotein cholesterol, total cholesterol, cognitive function score, and CESD-10 score in 2011

**Supplementary Table 2 The association between AIP and the risk of depression according to glucose metabolic states**

| Categories | Event, n (%) | Model 1 ^a^ | | Model 2 ^b^ | | Model 3 ^c^ | | P-interaction |
| --- | --- | --- | --- | --- | --- | --- | --- | --- |
|  |  | HR (95% CI) | P value | HR (95% CI) | P value | HR (95% CI) | P value |  |
|  | | | | | | | |  |
| **NGR** | | | | | | | | **0.022** |
| Per 1 unit increase | 327 (10.3%) | 1.44 (0.99-2.08) | 0.500 | 1.41 (0.98-2.03) | 0.067 | 1.51 (0.95-2.39) | 0.079 |  |
| Quartile 1 | 93 (9.5%) | Ref. |  | Ref. |  | Ref. |  |  |
| Quartile 2 | 91 (10.3%) | 1.09 (0.82-1.46) | 0.545 | 1.07 (0.80-1.43) | 0.646 | 1.25 (0.88-1.78) | 0.213 |  |
| Quartile 3 | 82 (10.6%) | 1.12 (0.83-1.51) | 0.452 | 1.08 (0.80-1.45) | 0.626 | 1.17 (0.80-1.69) | 0.419 |  |
| Quartile 4 | 61 (11.5%) | 1.22 (0.88-1.68) | 0.227 | 1.20 (0.87-1.66) | 0.268 | 1.26 (0.83-1.89) | 0.275 |  |
| **Pre-DM** | | | | | | | |  |
| Per 1 unit increase | 691 (20.3%) | 2.83 (2.29-3.50) | <0.001 | 2.71 (2.19-3.36) | <0.001 | 3.74 (2.75-5.10) | <0.001 |  |
| Quartile 1 | 87 (12.2%) | Ref. |  | Ref. |  | Ref. |  |  |
| Quartile 2 | 134 (16.2%) | 1.52 (1.16-1.99) | 0.002 | 1.50 (1.14-1.96) | 0.003 | 1.89 (1.37-2.62) | <0.001 |  |
| Quartile 3 | 199 (23.0%) | 2.25 (1.75-2.89) | <0.001 | 2.16 (1.68-2.78) | <0.001 | 2.84 (2.09-3.86) | <0.001 |  |
| Quartile 4 | 271 (29.6%) | 3.01 (2.37-3.84) | <0.001 | 2.86 (2.25-3.65) | <0.001 | 3.53 (2.61-4.78) | <0.001 |  |
| **DM** | | | | | | | |  |
| Per 1 unit increase | 293 (25.4%) | 2.08 (1.64-2.65) | <0.001 | 1.96 (1.54-2.51) | <0.001 | 2.72 (1.72-4.32) | <0.001 |  |
| Quartile 1 | 14 (8.9%) | Ref. |  | Ref. |  | Ref. |  |  |
| Quartile 2 | 38 (17.2%) | 2.03 (1.10-3.75) | 0.023 | 1.89 (1.02-3.49) | 0.043 | 1.72 (0.85-3.50) | 0.132 |  |
| Quartile 3 | 78 (26.8%) | 3.36 (1.90-5.93) | <0.001 | 3.08 (1.74-5.46) | <0.001 | 2.65 (1.37-5.13) | 0.004 |  |
| Quartile 4 | 163 (33.7%) | 4.40 (2.55-7.60) | <0.001 | 4.00 (2.31-6.91) | <0.001 | 3.75 (1.98-7.08) | <0.001 |  |

AIP: atherogenic index of plasma; CI: confidence interval; DM: diabetes mellitus; HR: hazard ratios; NGR: normal glucose regulation; Pre-DM: prediabetes mellitus.

^a^ Unadjusted model

^b^ Adjusted for age and gender

^c^ Adjusted for age, gender, body mass index, marital status; residence, educational level, health, smoking status, drinking status, chronic diseases, hypertension, low-density lipoprotein cholesterol, total cholesterol, cognitive function score, and CESD-10 score in 2011
